# NAC changes the course of cerebral small vessel disease in SHRSP and reveals new insights for the meaning of stases - a randomized controlled study

**DOI:** 10.1186/2040-7378-5-5

**Published:** 2013-04-15

**Authors:** Celine Zoe Bueche, Cornelia Garz, Siegfried Kropf, Daniel Bittner, Wenjie Li, Michael Goertler, Hans-Jochen Heinze, Klaus Reymann, Holger Braun, Stefanie Schreiber

**Affiliations:** 1 Department of Neurology, Otto-von-Guericke-University, Leipziger Strasse 44, Magdeburg, 39120, Germany; 2 Institute of Biometry and Medical Informatics, Otto-von-Guericke-University, Leipziger Strasse 44, Magdeburg, 39120, Germany; 3 Leibniz Institute for Neurobiology, Brenneckestrasse 6, Magdeburg, 39118, Germany; 4 German Center for Neurodegenerative Diseases (DZNE), Brenneckestrasse 6, Magdeburg, 39118, Germany

**Keywords:** Animal model, Blood–brain barrier, Cerebral microbleed, Cerebral small vessel disease, von Willebrand factor

## Abstract

**Background:**

N-Acetylcystein (NAC) reduces the reperfusion injury and infarct size in experimental macroangiopathic stroke. Here we now investigate the impact of NAC on the development of the histopathology of microangiopathic cerebrovascular disease including initial intravasal erythrocyte accumulations, blood–brain-barrier (BBB)-disturbances, microbleeds and infarcts.

**Methods:**

Spontaneously Hypertensive Stroke-Prone Rats (SHRSP) were treated with NAC (12 mg/kg body weight, daily oral application for three to 30 weeks) and compared to untreated SHRSP. In all rats the number of microbleeds, thromboses, infarcts and stases were quantified by HE-staining. Exemplary brains were stained against von Willebrand factor (vWF), IgG, Glutathione and GFAP.

**Results:**

NAC animals exhibited significant more microbleeds, a greater number of vessels with BBB-disturbances, but also an elevation of Glutathione-levels in astrocytes surrounding small vessels. NAC-treatment reduced the numbers of thromboses, infarcts and arteriolar stases.

**Conclusions:**

NAC reduces the frequency of thromboses and infarcts to the expense of an increase of small microbleeds in a rat model of microangiopathic cerebrovascular disease. We suppose that NAC acts via an at least partial inactivation of vWF resulting in an insufficient sealing of initial endothelial injury leading to more small microbleeds. By elevating Glutathione-levels NAC most likely exerts a radical scavenger function and protects small vessels against extended ruptures and subsequent infarcts. Finally, it reveals that stases are mainly caused by endothelial injuries and restricted thromboses.

## Introduction

It has been repeatedly demonstrated, that treatment with N-Acetylcystein (NAC), a precursor of Glutathione and an antioxidant also used as mucolyticum, reduces the reperfusion injury and infarct size in animal models of transient and permanent middle cerebral artery occlusion mimicking acute macroangiopathic cerebrovascular disease [1-4]. It is assumed that this neuroprotective effect of NAC is mainly caused by its anti-inflammatory potential [1,2,4]. In addition to that NAC also interferes with the coagulation cascade [5-7].

Recently we described in Spontaneously Hypertensive Stroke-Prone Rats (SHRSP) the cerebral small vessel disease (CSVD) as a cascade starting from capillary and arteriolar erythrocyte accumulations we referred to as stases and blood–brain-barrier (BBB)-damage leading via microbleeds to secondary thromboses and infarctions [8,9]. Now, we were interested whether a long-term treatment of these rats with NAC influences the development of CSVD.

NAC surprisingly significantly increased the frequency of small microbleeds at younger ages but on the other hand lowered the number of greater microbleeds, secondary thromboses and infarcts later on. We explain this double edged action of NAC by an inactivation of the von Willebrand factor (vWF) and downregulation of local coagulation on the one hand and an increased Glutathione in astrocyte end-feet of the BBB on the other hand. Finally, our data reveal that stases are most likely caused by a local coagulation occurring in reaction to endothelial injuries of small vessels.

## Material and methods

### Animals and treatment

Animal procedures were approved by the Animal Care Committee of Sachsen-Anhalt. The rats were housed with a natural light–dark cycle and allowed to access water and food ad libitum; no salt-loaded diet was applied.

Forty six male SHRSP (Charles River Laboratories International Inc., Wilmington, MA, USA) were treated uniformly starting from an age of 15 weeks with NAC (daily application, dosage 12 mg/kg body weight per day, given orally via drinking water) for different time periods until their perfusion at ages of 18 to 22 (n=8), 24 to 26 (n=8), 28 (n=9), 31 to 32 (n=6), 34 to 36 (n=6) and 40 to 44 (n=9) weeks (w). Fifty nine male SHRSP at corresponding ages (18 to 22 w n=8, 24 to 26 w n=8, 28 to 30 w n=9, 31 to 32 w n=9, 34 to 36 w n=13, 40 to 44 w n=12) served as a non-treated control group. The allocation of the rats to the treated or non-treated group was randomized.

### Histology and immunohistochemistry

Rats were transcardially perfused (120 mL phosphate buffered saline (PBS), 120 mL 4% paraformaldehyde (PFA)). The brains were removed, fixed in 4% PFA (48 hours), placed for cryoprotection into 30% sucrose (6 days), and frozen in methylbutane at −80°C. Coronal slices of the whole brain were prepared using a cryotoma (Leica, Nussloch, Germany). From the frontal to the occipital pole there were 10 to 11 sectional planes per animal, whereas the first sectional plane was remote 930 μm from the frontal pole. The distance between each sectional plane was about 1 mm. Chosen a continuous distance of 840 μm from the respect forward sectional plane three slices per brain sectional plane and, thus, thirty one to 33 slices per animal of all 105 SHRSP were stained with Hematoxylin-Eosin (HE).

Three slices per brain (frontal sectional plane with basal ganglia and without hippocampus, mid sectional plane with anterior/middle hippocampus and basal ganglia, posterior sectional plane with hippocampus and without basal ganglia) of exemplary animals (treated group n= 6, non-treated group n=7) at different stages of age were stained immunohistochemistally by STL-FITC (solanum tuberosum lectin as endothelial marker, [10]), and antibodies against vWF, Glutathione, GFAP (glial fibrillary acidic protein, marker for glia cells) and IgG. In short, the immunohistochemistry was performed as follows: blocking with 0.1 mol/L PBS, 0.5% TritonX, and 10% donkey serum (Sigma, St Louis, MO, USA) was followed by staining with STL-FITC (lectin from potato, Vector Laboratories, Burlingame, CA, USA; 1:500) and anti-vWF (rabbit, polyclonal, Abcam, Cambridge Science Park, Cambridge, UK; 1:200) or anti-Glutathione (mouse, monoclonal, Abcam, Cambridge Science Park, Cambridge, UK; 1:150) and anti-GFAP (chicken, polyclonal, Chemicon/ Milipore, Schwalbach 1:250) overnight at 4°C in PBS containing 5% donkey serum; and then incubated with the secondary antibodies Cy3-donkey-anti-rabbit-IgG (polyclonal, detection of vWF; 1:500), Cy3-donkey-anti-mouse-IgG (polyclonal, detection of Glutathione; 1:500), Cy5-donkey-anti-rat-IgG (polyclonal, detection of IgG; 1:500) or Cy5-donkey-anti-chicken-IgG (polyclonal, detection of GFAP, 1:500) for 2 hours at room temperature; finally DAPI staining (20 minutes, room temperature) was performed. After dehydration with increasing concentrations of alcohol, slices were mounted on slides with Histomount.

### Quantification

Using light microscopy (Leica DMR) in the HE sections of all SHRSP (31 sections per brain and animal, analysis of the whole section including all brain areas) the following parameters were blinded quantified separately: small microbleeds < 150 μm, larger microbleeds >150 μm, thromboses. In 25 exemplary animals (treated group n=15, non-treated group n=10) of different ages the extent of erythrocyte accumulations was assessed separately for capillaries (diameter 5 to 20 μm [11]) and arterioles (diameter > 20 to 65 μm [12]) in all brain regions: 0=no accumulated erythrocytes per field of view (FoV), 1= < 5 vessels with accumulated erythrocytes per FoV, 2= 5–15 vessels with accumulated erythrocytes per FoV, 3= more than 15 vessels with accumulated erythrocytes per FoV. Ten sections (50 FoV) per brain and animal were analyzed.

STL-, vWF- and IgG-positive as well as STL- and Glutathione-positive vessels in all brain regions were quantified in 3 sections (52 to 72 counting boxes) per brain and animal. The ratios of vWF/STL-positive, Glutathione/STL-positive, IgG/STL-positive vessels as well as the vWF and IgG Colocalization/STL were calculated for each animal.

### Statistics

Using the nonparametric Mann–Whitney Test all quantified parameters (bleeds, thromboses, erythrocyte accumulations, vWF/STL-positive vessels, IgG/STL-positive vessels, Glutathione/STL-positive vessels, vWF and IgG Colocalization/STL) were compared across the age groups of the treated and non-treated group. Using Spearman’s correlation coefficient we separately investigated the association between all quantified parameters in both (treated, non-treated) animal groups. To assess a possible age-dependency of all quantified parameters we used an univariate analysis of variance with group (treated, non-treated rats) as fixed factor and age as covariate. P-values equal or less than 0.05 were deemed to be statistically significant.

## Results

### Microbleeds

**Figure 1:**
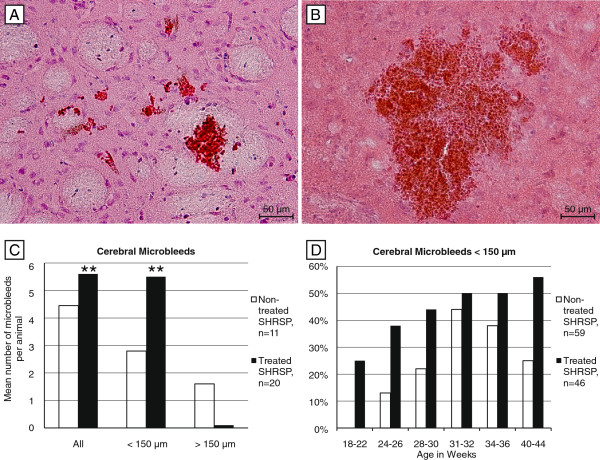
**Microbleeds in treated and non-treated SHRSP.** Microbleeds in the basal ganglia of a 40 weeks old treated (<150 μm, **A**) and a 31 weeks old non-treated SHRSP (> 150 μm, **B**). The histograms of all animals with cerebral microbleeds (n=31) show significant more microbleeds per animal in the treated group (**C**) caused by significant more small microbleeds (< 150 μm) per animal (**A** &**C**) and age (**D**) in the treated group. In contrast, compared to the non-treated group the treated rats were less often affected by microbleeds > 150 μm (**C**). **A**, **B** HE staining, basal ganglia, magnification 200, ** p ≤ 0.05.

Both animal groups exhibited microbleeds, in the treated rats 20 of 46 animals (44%) and in the non-treated rats 11 of 59 (19%) animals were affected. Comparing both groups the treated rats had a significant higher risk for the development of microbleeds in general (p=0.001), for the development of small microbleeds (< 150 μm, p=0.001; Figure 1A-C) and exhibited significant more small microbleeds per age group and at younger ages (Figure 1D). Although there were no statistical differences between the groups concerning bleeds > 150 μm (p=0.720), the treated animals were less often affected by those larger microbleeds (Figure 1C).

### Thromboses and infarcts

**Figure 2:**
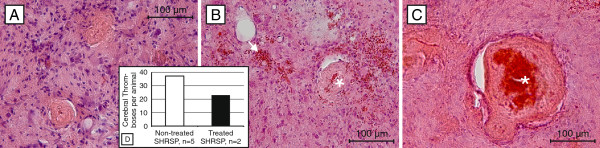
**Thromboses and infarcts in treated and non-treated SHRSP.** Thromboses (**A**-**C**) with associated luminal hemorrhages (asterisk, **B** &**C**) and adjacent - to some extent hemorrhagic (arrow, **B**) - tissue infarcts (**A**-**C**). **D** - The histogramms of all animals with cerebral thromboses (n=7) show less thromboses per animal in the treated group. **A**-**C** HE staining, **A** – 31 weeks old non-treated SHRSP, basal ganglia, **B** – 42 weeks old treated SHRSP, cortex, **C** – 31 weeks old non-treated SHRSP, cortex; magnification 200.

In the treated group 2 of 46 SHRSP (4%) and in the non-treated group 5 of 59 animals (9%) exhibited thromboses with associated cerebral infarcts (Figure 2A-C). The occurrence of thromboses was significantly age-dependent in the non-treated animals (p=0.049). Although there were obviously less infarcts and thromboses in the treated rats, those parameters did not differ statistically significantly between the groups (p=0.182; Figure 2D).

### Erythrocyte accumulations

**Figure 3:**
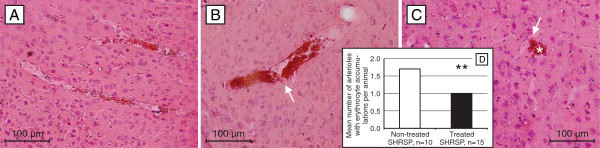
**Arteriolar erythrocyte accumulations in treated and non-treated SHRSP.** Arteriolar erythrocyte accumulations in a 18 weeks old (**A**) and in a 36 weeks (**B**) non-treated animal and 32 weeks (**C**) old treated animal. Comparing both groups, the treated and the non-treated SHRSP, those arteriolar erythrocyte accumulations were less detectable in the treated animals (**D**). Note the initiating (arrow, **B**) and complete (arrow, **C**) diapedesis of the intravasal accumulated erythrocytes throughout the damaged small vessel wall leading to microbleeds in the adjacent parenchyma (asterisk, **C**). **A**-**C** HE staining, cortex, magnification 200, ** p ≤ 0.05.

Erythrocyte accumulations occur first in the capillary bed and then in arterioles later on [9]. The ratio between both forms of stases was changed by NAC-treatment. Untreated rats developed 57% of stases in capillaries and 43% in arterioles, whereas after NAC treatment 67% of stases were found in capillaries and only 33% were located in arterioles. Age-controlled analysis revealed significantly less arteriolar erythrocyte aggregations in the treated rats compared to the non-treated group (p=0.05; Figure 3A-D), whereas there were no significant group differences regarding the number of capillary erythrocyte accumulations (p=0.462).

### Von Willebrand factor and IgG

**Figure 4:**
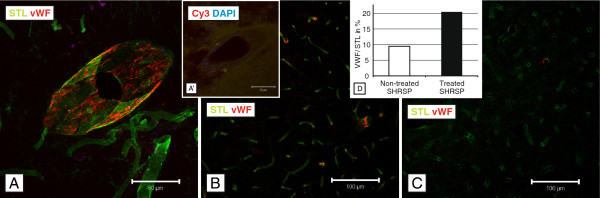
**STL- (green) and anti-vWF- (red) staining in treated and non-treated SHRSP.** Comparing treated (**B**, 24 weeks old, cortex, magnification 200) and non-treated (**C**, 24 weeks old, cortex, magnification 200) animals an increased number of vWF-positive small vessels is detectable in the treated rats (**D**, histogram, y-axis with mean ratio of vWF-/STL-positive vessels in%). **A** - vWF threads stick to the inner vessel wall indicating small endothelial injuries (24 weeks old treated SHRSP, basal ganglia, magnification 400). **A`**- Negative control for vWF (Cy3 donkey x rabbit). DAPI - 4',6-diamidino-2-phenylindole, fluorescent stain for DNA detection.

**Figure 5:**
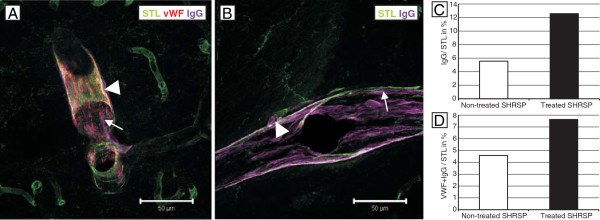
**STL- (green), anti-vWF- (red) and anti-IgG (magenta) staining in treated and non-treated SHRSP.** IgG, indicating BBB disturbances, is localized in the small vessel walls (arrow, **A** &**B**), in the adjacent perivascular parenchyma (arrowhead, **A** &**B**) and colocalized with vWF (**A**). Comparing both groups, the treated one exhibited more IgG-positive vessels (**C**, histogram, y-axis with mean ratio of IgG-/STL-positive vessels in %) and more colocalization of IgG and vWF (**D**, histogram, y-axis with mean ratio of vWF- and IgG-/STL-positive vessels in %). **A** – 26 weeks old treated animal, basal ganglia, magnification 400, **B** – 26 weeks old non-treated SHRSP, basal ganglia, magnification 400.

The vWF was adherent at the small vessel walls (Figure 4A-C) or was detected within the lumen of the small vessels where it was often associated with the occurrence of intravasal erythrocyte accumulations [8]. IgG, indicative for BBB-disturbances [10,13], was found to be accumulated in the small vessel walls as well as in the parenchyma adjacent to the capillaries and arterioles (Figure 5A & B; [8,9]). In both animal groups vWF and IgG were predominantly found in the arterioles. The treated group exhibited nearly significantly more vWF- and IgG-immunoreactivity compared to the non-treated group (p=0.084 respectively, age-controlled analysis; Figure 4B, C & D, Figure 5C). Additionally, the treated animals exhibited a nearly significantly more frequent colocalization of vWF and IgG in the small vessel walls (p=0.084, age-controlled analysis; Figure 5A & D).

### Glutathione

**Figure 6:**
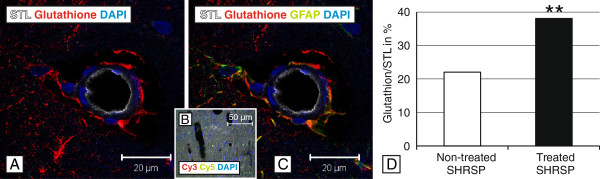
**STL- (white), Glutathione- (red) and GFAP- (green) immunohistochemistry in a treated SHRSP.** Glutathione is produced by astrocytes juxtapositioned to the small vessels’ endothelial cells (**A** &**C**). Comparing both groups, the treated one exhibited significant more Glutathione-positive vessels surrounded by astrocytes (**D**, histogram, y-axis with mean ratio of Glutathione-positive vessels in %). **B** – Negative control for Glutathione (Cy3 donkey x mouse) and GFAP (Cy5 donkey x chicken). DAPI - 4',6-diamidino-2-phenylindole, fluorescent stain for DNA detection. **A**, **C** hippocampal arteriole of a 26 weeks old SHRSP, magnification 630, ** p ≤ 0.05.

In both groups, the treated and non-treated one, Glutathione was expressed in GFAP-positive astrocytes adjacent to the small vessel wall suggesting a derive of Glutathione by astrocytes juxtapositioned to endothelial cells and maintaining the BBB (Figure 6A & C; [14]). Quantification and statistical analysis revealed a significant increase of Glutathione within astrocytic end-feet encircling the small vessel walls of the NAC group (p=0.000, age-controlled analysis Figure 6D).

## Discussion

In the present study we investigated the influence of chronic NAC application on the development of CSVD in SHRSP. Treatment started at 15 weeks of age and lasted between three and about 30 weeks. Our initial aim was to investigate whether NAC is capable to decelerate the development of CSVD in SHRSP previously described by our group [9]. Therefore animals of different ages were checked by HE staining for the presence of erythrocyte accumulations, microbleeds and infarcts characterized by necrotic tissue containing hemorrhages and thromboses.

An increased number of microbleeds was surprisingly the most striking difference in the NAC group. Treated animals exhibited microbleeds at younger ages and the number of microbleeds per animal was significantly higher compared to the control group. This increase was exclusively accounted for by microbleeds smaller than 150 μm. In contrast untreated animals developed microbleeds at later ages and the overall frequency was in this group significantly lower. However, it became obvious that the control animals had a higher proportion of microbleeds larger than 150 μm.

According to our previous investigations microbleeds are part of a pathological cascade of CSVD initiated by capillary and arteriolar erythrocyte accumulations we referred to as stases, followed by those microbleeds with reactive thromboses and associated infarcts [9]. Therefore, we were also interested in the impact of NAC treatment on the number of animals developing thromboses and infarcts on the one hand and on the number of stases occurring per animal on the other hand. Interestingly, infarcts and associated thromboses were less often detectable in the treated animals (4%) compared to the non-treated group (9%).

Moreover, comparing both groups the medicated rats exhibited less arteriolar stases. That means that NAC treatment increases microbleeds on the one hand but reduces the initial stases and the later occurring thromboses and associated infarcts on the other hand.

Recently we have shown that stases mark small vessel wall segments with deposits of plasma proteins like IgG. Moreover, we could demonstrate that stases are associated with an activated coagulatory state indicated by vWF-immunoreactivity within the accumulations of erythrocytes [8]. Our present data show, that NAC treatment leads to an increased detectability of those IgG deposits as well as to an increase of vWF-positive small vessels.

vWF is a key sensor for endothelial injuries leading to an exposure of the subendothelial matrix. Endothelial injuries combined with the physiological intravasal shear stress cause a conformation change of the normally compact vWF protein into elongated multimers [6,15]. The elongated form of vWF exposures multiple binding sites for platelets and optimizes the adhesion between aggregated thrombocytes and fibrin resulting in thrombus formation within the injured vessel [6,16-18]. We now suppose that this activated coagulation is also the main reason for the formation of stases by sticking of erythrocytes to the thrombotic material. Therefore, stases (visible in HE staining) seem to indicate small vessel wall segments with BBB breakdown.

Two aspects of this permanent occurring coagulation activation are critical: (i) Emerged endothelial injuries have to be sealed immediately and properly, otherwise plasma proteins are leaking into the subendothelial space. (ii) The triggered thrombosis has to be restricted in order to avoid vessel obstruction. The latter is realized by the protease ADAMST13 decreasing the multimer size of vWF by cleaving it within its A2 domain and secondly by reducing intra-multimer disulfide bonds. Both activities lead to a reduction of vWF multimer-size and its pro-thrombotic function [6,19].

Such a cleavage of vWF disulfide bonds is also performed by NAC [6]. Thus, through the long lasting application of NAC the vWF balance has been shifted to shorter multimers exhibiting a lower number of binding sites for platelets. This increased number of vWF cleavage products and a probable compensatory augmented production could facilitate an increased effectiveness of antibody binding after NAC treatment. This explains the greater number of vWF-positive small vessels in the NAC group. Most importantly, the functional consequence would be an imperfect sealing of many endothelial leakage sites and a higher rate of plasma protein deposits (i.e. IgG). This is supported by a higher rate of colocalizations of vWF and IgG immunoreactivity found in the NAC group.

Ongoing deposits of plasma proteins into the vessel wall cause on their part an increasing BBB damage of the small vessel wall, finally resulting in a vessel wall rupture with consecutive microbleeds. Since NAC only attenuates and not extinguishes the prothrombotic function of vWF [6] only a limited amount of blood is leaking into the parenchyma. Therefore, the majority of emerging microbleeds is smaller than 150 μm. Interestingly, the number of arterioles with accumulated erythrocytes is in NAC animals reduced. This indicates that the development of stases obviously depends on the extent of local thrombus formation.

Despite the greater number of small microbleeds, the NAC treatment decelerates the course of CSVD in SHRSP since untreated animals develop larger microbleeds (>150 μm), more reactive thromboses and related to that more infarcts. This can be explained by the second, cytoprotective effect of NAC.

First the situation in control animals: Untreated rats develop endothelial injuries with the same magnitude as NAC treated. However, due to their greater number of fully activated vWF multimers these leakage sites are sealed more effectively with the consequence that in control animals plasma-proteins are deposited more rarely, BBB damages are avoided more effectively and consequently small microbleeds occur less frequently. Contrary, the number of arteriolar stases is in control animals higher, since the development of them, as already mentioned, seems to be dependent on the formation of local thrombi at sites with endothelial injuries.

However, with increasing age the number of leakage sites increases permanently [9] and more and more arterioles are afflicted. Despite an effective sealing by the coagulatory system arteriolar sites with fast developing BBB damage remain brashly [20]. On site stases emerging together with the coagulation might further deteriorate that vascular wall damage by accumulating iron-containing hemoglobin products [14]. All this is accompanied by a chronic elevated blood pressure in SHRSP [21]. Both circumstances together can cause vessel wall ruptures of arterioles with the consequence of greater microbleeds in control rats (> 150 μm).

This situation is changed by the cytoprotective action of NAC. NAC increases the Glutathione-production especially in astrocytes surrounding with their end-feet the endothelial cells and by this composing a part of the BBB [14,22]. Although NAC downregulates the coagulation system and through this provokes small microbleeds, an elevated Glutathione level simultaneously protects the endothelium and smooth muscle cells of capillaries and arterioles by its radical scavenger function [14,23]. Moreover, it is conceivable that the greater number of small microbleeds in NAC animals causes a local pressure relief in more proximal arterioles. All these factors together could explain why chronic NAC treatment reduces the number of arteriolar ruptures. Consequently, subsequent larger microbleeds and connected with them reactive thromboses and infarcts are also reduced [1-4].

## Conclusion

The chronic application of NAC seems to decelerate the course of CSVD in SHRSP mediated by a cytoprotective effect of an increased Glutathione allocation. Treated animals develop a reduced number of larger microbleeds and following them reactive thromboses and infarcts. This effect however, occurs at the expense of a greater number of small microbleeds emerging already in younger rats. We assume that the chronic downregulation of the coagulation system mainly by inhibition of the vWF -function is the most likely explanation for that. Further, this study reveals that a restricted thrombus formation at tiny injuries of the endothelium most likely induces the generation of stases as the first visible vascular peculiarity in SHRSP.

Transferring our data to the clinical setting, NAC, often used for mucolytic therapy in post-stroke pneumonia, might cause more small microbleeds in patients with degenerative cerebral small vessel wall changes. On the other hand, NAC might be evaluated as an additional agent to prevent cerebral thromboses and associated infarcts in patients confronted with the risk of CSVD.

## Abbreviations

BBB: Blood brain barrier; CSVD: Cerebral small vessel disease; Cy: Cyanine; DAPI: 4',6-diamidino-2-phenylindole; FoV: Field of view; GFAP: Glial fibrillary acidic protein; HE: Hematoxylin-Eosin; NAC: N-Acetylcystein; PBS: Phosphate buffered saline; PFA: Paraformaldehyde; SHRSP: Spontaneously hypertensive stroke-prone rats; STL-FITC: Solanum tuberosum lectin fluorescein isothiocyanate; vWF: von Willebrand factor.

## Competing interests

All authors declare that they have no competing interest.

## Authors’ contributions

CB, CG, HB and SS collected, analyzed and interpreted the data. DB and WL were involved in data interpretation. SK performed the statistical analyses. CB, CG, SS, HB, HJH, MG and KR were involved in planning the study design and contributed to the writing of the final version of the manuscript. All authors read and approved the final manuscript.
